# Supplementary material for: Retrospective Evaluation of Method of Treatment, Laboratory Findings, and Concurrent Diseases in Dairy Cattle Diagnosed with Left Displacement of the Abomasum during Time of Hospitalization
Source: Animals (Basel). 2022 Jun 27;12(13):1649. doi: 10.3390/ani12131649 (PMC9265103; doi:10.3390/ani12131649)
Supplement: Supplementary file 1 [file animals-12-01649-s001.zip › animals-1744860-supplementary.pdf]

Table S1: Listing of analgesic and oral treatment (components) in 209 animals submitted to abomasal rolling for treatment of left displacement of the abomasum at the Clinic for Ruminants with Ambulatory and Herd Health Services from 2009 to 2019. Treatment is variable and was dependent on the surgeon, especially considering the administration of sodium sulfate. The amount of water used for the oral drench was rarely documented. Number of animal is abbreviated as Animal.

Animal	Analgesic treatment	Oral drench
**1**	- Metamizole<br>- Flunixine meglumine	- Propylene glycol<br>- Sodium Sulfate<br>- Vitamin E/Selenium<br>- KCl
**2**	- Ketoprofen	- Propylene glycol<br>- Vitamin E/Selenium<br>- KCl
**3**	- Metamizole<br>- Flunixine meglumine	- Vitamin E/Selenium<br>- KCl
**4**	- Flunixine meglumine	- None
**5**	- Ketoprofen	- None
**6**	- Flunixine meglumine	- Propylene glycol<br>- Vitamin E/Selenium<br>- KCl<br>- NaBic
**7**	- Metamizole	- None
**8**	- Flunixine meglumine	- Propylene glycol<br>- Vitamin E/Selenium<br>- Ca
**9**	- Meloxicam	- None
**10**	- Flunixine meglumine<br>- Metamizol	- Propylene glycol<br>- Vitamin E/Selenium<br>- KCl<br>- NaBic
**11**	- Flunixine meglumine	- None
**12**	- Flunixine meglumine<br>- Metamizol	- Propylene glycol<br>- Vitamin E/Selenium<br>- KCl
**13**	- Meloxicam	- None
**14**	- Ketoprofen	- Propylene glycol<br>- Vitamin E/Selenium<br>- KCl<br>- Ca
**15**	- None	- None
**16**	- Flunixine meglumine	- Propylene glycol<br>- KCl<br>- Ca
**17**	- Metamizole<br>- Meloxicam	- Propylene glycol<br>- Vitamin E/Selenium

		- KCl<br>- Sodium sulfate
**18**	- Metamizole<br>- Flunixine meglumine	- None
**19**	- Metamizole<br>- Meloxicam	- None
**20**	- None	- Sodium sulfate<br>- Vitamin E/Selenium<br>- KCl<br>- Ca
**21**	- Metamizole<br>- Flunixine meglumine	- None
**22**	- Metamizole<br>- Flunixine meglumine	- Propylene glycol<br>- Vitamin E/Selenium<br>- KCl<br>- Ca
**23**	- Flunixine meglumine	- Vitamin E/Selenium<br>- KCl
**24**	- None	- Propylene glycol<br>- KCl<br>- Ca
**25**	- Flunixine meglumine	- Vitamin E/Selenium<br>- KCl<br>- Ca
**26**	- Ketoprofen	- Propylene glycol<br>- KCl<br>- Ca
**27**	- Flunixine meglumine<br>- Metamizole	- Propylene glycol<br>- Vitamin E/Selenium<br>- KCl<br>- Ca
**28**	- Flunixine meglumine	- Propylene glycol<br>- Vitamin E/Selenium<br>- KCl<br>- Ca
**29**	- Metamizole	- None
**30**	- Flunixine meglumine<br>- Metamizol	- Vitamin E/Selenium
**31**	- Metamizole	- None
**32**	- Meloxicam	- Propylene glycol<br>- Vitamin E/Selenium<br>- KCl
**33**	- Ketoprofen	- Propylene glycol<br>- Vitamin E/Selenium<br>- KCl<br>- Ca

**34**	- Meloxicam	- KCl
**35**	- Meloxicam	- None
**36**	- Ketoprofen	- None
**37**	- Ketoprofen	- None
**38**	- Metamizole	- None
**39**	- Flunixine meglumine<br>- Metamizole	- None
**40**	- Ketoprofen	- Propylene glycol<br>- Sodium Sulfate<br>- Vitamin E/Selenium<br>- KCl<br>- Ca
**41**	- Meloxicam	- None
**42**	- Flunixine meglumine	- None
**43**	- Meloxicam	- Propylene glycol<br>- Vitamin E/Selenium<br>- KCl<br>- Ca
**44**	- Ketoprofen	- None
**45**	- Meloxicam	- None
**46**	- Flunixine meglumine	- None
**47**	- Flunixine meglumine<br>- Metamizole	- Propylene glycol<br>- Sodium Sulfate<br>- Vitamin E/Selenium<br>- KCl
**48**	- Meloxicam	- None
**49**	- Flunixine meglumine	- Propylene glycol<br>- Vitamin E/Selenium<br>- Sodium sulfate
**50**	- Flunixine meglumine	- None
**51**	- Flunixine meglumine	- Propylene glycol<br>- Vitamin E/Selenium<br>- NadPh
**52**	- Ketoprofen	- None
**53**	- Meloxicam	- None
**54**	- Ketoprofen	- Propylene glycol<br>- KCl<br>- Ca
**55**	- Meloxicam	- KCl<br>- NaCl
**56**	- Flunixine meglumine	- Sodium Sulfate<br>- Vitamin E/Selenium<br>- KCl
**57**	- Ketoprofen<br>- Metamizole	- Sodium Sulfate<br>- Vitamin E/Selenium<br>- KCl

		- Ca<br>- NaCl
**58**	- None	- None
**59**	- Meloxicam	- Propylene glycol<br>- KCl<br>- Ca
**60**	- Ketoprofen	- Propylene glycol<br>- Vitamin E/Selenium<br>- NadPh
**61**	- Flunixine meglumine	- Propylene glycol<br>- Vitamin E/Selenium<br>- KCl
**62**	- Flunixine meglumine	- Propylene glycol<br>- Vitamin E/Selenium<br>- KCl<br>- Ca
**63**	- Meloxicam	- Propylene glycol<br>- Vitamin E/Selenium<br>- KCl<br>- Ca
**64**	- Flunixine meglumine	- Propylene glycol<br>- Sodium Sulfate<br>- Vitamin E/Selenium<br>- KCl
**65**	- Flunixine meglumine	- Propylene glycol<br>- Sodium Sulfate<br>- Vitamin E/Selenium<br>- KCl
**66**	- Dexamethasone	- Propylene glycol<br>- KCl
**67**	- Ketoprofen	- None
**68**	- Flunixine meglumine	- Propylene glycol<br>- Sodium Sulfate<br>- KCl<br>- Ca
**69**	- Ketoprofen	- None
**70**	- Flunixine meglumine	- Propylene glycol<br>- Vitamin E/Selenium<br>- KCl
**71**	- Ketoprofen	- Propylene glycol<br>- Vitamin E/Selenium<br>- KCl<br>- NaBic
**72**	- Ketoprofen	- Propylene glycol<br>- Vitamin E/Selenium<br>- NaCl

		- NaBic
**73**	- Ketoprofen	- Propylene glycol<br>- Vitamin E/Selenium<br>- KCl<br>- Ca
**74**	- Metamizole<br>- Dexamethasone	- Propylene glycol<br>- KCl<br>- Ca<br>- NaCl
**75**	- Flunixine meglumine	- Vitamin E/Selenium<br>- KCl<br>- NadPh
**76**	- Flunixine meglumine	- Vitamin E/Selenium<br>- KCl
**77**	- Ketoprofen	- Propylene glycol<br>- Sodium Sulfate<br>- KCl<br>- Ca<br>- NaBic
**78**	- Flunixine meglumine	- None
**79**	- Flunixine meglumine	- None
**80**	- Flunixine meglumine	- None
**81**	- Flunixine meglumine	- Propylene glycol<br>- Sodium Sulfate<br>- KCl<br>- Ca<br>- NaBic
**82**	- Flunixine meglumine	- Sodium Sulfate<br>- KCl<br>- Ca<br>- NaBic
**83**	- Ketoprofen	- Propylene glycol<br>- 63Vitamin E/Selenium<br>- KCl<br>- Ca
**84**	- Metamizole	- Propylene glycole<br>- Vitamin E/Selenium<br>- KCl<br>- NaCl
**85**	- Flunixine meglumine	- Vitamin E/Selenium<br>- KCl<br>- Ca
**86**	- Flunixine meglumine	- Vitamin E/Selenium<br>- KCl
**87**	- Flunixine meglumine	- None
**88**	- Ketoprofen	- None

**89**	- Flunixine meglumine	- None
**90**	- Flunixine meglumine	- Propylene glycol<br>- Vitamin E/Selenium<br>- KCl
**91**	- Flunixine meglumine	- Propylene glycol<br>- Sodium sulfate<br>- Vitamin E/Selenium<br>- KCl<br>- Ca
**92**	- Flunixine meglumine	- None
**93**	- Flunixine meglumine	- Propylene glycol
**94**	- Ketoprofen	- None
**95**	- Flunixine meglumine	- None
**96**	- Flunixine meglumine<br>- Metamizole	- Propylene glycol<br>- Vitamin E/Selenium<br>- KCl<br>- Ca
**97**	- Flunixine meglumine	- Propylene glycol<br>- Vitamin E/Selenium<br>- KCl<br>- Ca
**98**	- Flunixine meglumine	- Propylene glycol<br>- Sodium Sulfate<br>- Vitamin E/Selenium
**99**	- Flunixine meglumine	- None
**100**	- Flunixine meglumine	- Propylene glycol<br>- Sodium Sulfate<br>- Vitamin E/Selenium<br>- KCl
**101**	- None	- None
**102**	- Ketoprofen	- Propylene glycol<br>- Sodium Sulfate<br>- KCl<br>- Ca
**103**	- Ketoprofen	- None
**104**	- Flunixine meglumine	- None
**105**	- None	- None
**106**	- Flunixine meglumine	- None
**107**	- Flunixine meglumine	- None
**108**	- None	- None
**109**	- Flunixine meglumine	- Sodium Sulfate<br>- Vitamin E/Selenium<br>- KCl
**110**	- Flunixine meglumine	- Vitamin E/Selenium<br>- NadPh
**111**	- Flunixine meglumine	- Propylene glycol

		- Vitamin E/Selenium<br>- KCl<br>- NadPh
**112**	- Flunixine meglumine	- Propylene glycol<br>- Sodium Sulfate<br>- Vitamin E/Selenium<br>- KCl<br>- NadPh
**113**	- Ketoprofen	- None
**114**	- Flunixine meglumine	- Propylene glycol<br>- Vitamin E/Selenium<br>- KCl
**115**	- Flunixine meglumine	- Propylene glycol<br>- Vitamin E/Selenium<br>- KCl<br>- Ca
**116**	- None	- Vitamin E/Selenium<br>- KCl<br>- Ca
**117**	- Flunixine meglumine	- None
**118**	- Ketoprofen	- Vitamin E/Selenium<br>- Ca
**119**	- None	- KCl
**120**	- Flunixine meglumine	- Propylene glycol<br>- Vitamin E/Selenium<br>- KCl<br>- Ca
**121**	- Flunixine meglumine	- Propylene glycol<br>- Vitamin E/Selenium<br>- KCl<br>- NaBic<br>- MgO
**122**	- None	- Vitamin E/Selenium<br>- KCl<br>- Ca
**123**	- Ketoprofen	- None
**124**	- Meloxicam	- Sodium sulfate<br>- Vitamin E/Selenium<br>- KCl<br>- Ca
**125**	- None	- None
**126**	- Ketoprofen	- None
**127**	- Meloxicam	- Vitamin E/Selenium<br>- KCl
**128**	- Meloxicam	- Propylene glycol<br>- Vitamin E/Selenium

		- KCl<br>- Ca
**129**	- Flunixine meglumine	- Propylene glycol<br>- Vitamin E/Selenium<br>- KCl<br>- Ca
**130**	- Meloxicam	- None
**131**	- Ketoprofen	- None
**132**	- Ketoprofen	- None
**133**	- Ketofprofen	- Sodium Sulfate<br>- Vitamin E/Selenium<br>- KCl
**134**	- Flunixine meglumine	- None
**135**	- Flunixine meglumine	- Propylene glycol<br>- Vitamin E/Selenium<br>- KCl
**136**	- Flunixine meglumine	- Vitamin E/Selenium<br>- KCl<br>- Ca
**137**	- Flunixine meglumine	- Propylene glycol<br>- KCl<br>- Ca
**138**	- Flunixine meglumine	- None
**139**	- Flunixine meglumine	- None
**140**	- Flunixine meglumine	- None
**141**	- Flunixine meglumine	- None
**141**	- Flunixine meglumin	- Vitamin E/Selenium<br>- KCl<br>- Ca
**142**	- Flunixine meglumine	- Propylene glycol<br>- Vitamin E/Selenium<br>- KCl<br>- Ca
**143**	- None	- None
**144**	- Meloxicam	- Vitamin E/Selenium
**145**	- Flunixine meglumine	- None
**146**	- Meloxicam	- None
**147**	- Flunixine meglumine	- Propylene glycol<br>- Vitamin E/Selenium<br>- KCl
**148**	- Meloxicam	- None
**149**	- Ketoprofen	- Vitamin E/Selenium<br>- KCl
**150**	- Ketoprofen	- None
**151**	- Flunixine meglumine	- None
**152**	- Flunixine meglumine	- None

**153**	- Flunixine meglumine	- None
**154**	- None	- None
**155**	- Ketoprofen	- None
**156**	- Flunixine meglumine	- None
**157**	- Meloxicam	- None
**158**	- Flunixine meglumine	- None
**159**	- None	- None
**160**	- Meloxicam	- None
**161**	- Ketoprofen	- None
**162**	- Flunixine meglumine	- Propylene glycol<br>- Sodium Sulfate<br>- Vitamin E/Selenium<br>- KCl<br>- Ca
**163**	- Dexamethasone<br>- Flunixine meglumine	- None
**164**	- Meloxicam	- None
**165**	- Flunixine meglumine	- Vitamin E/Selenium<br>- KCl
**166**	- Ketoprofen	- None
**167**	- Meloxicam	- Vitamin E/Selenium<br>- Ca
**168**	- Ketoprofen	- None
**169**	- Meloxicam	- None
**170**	- Flunixine meglumine	- None
**171**	- Flunixine meglumine	- None
**172**	- Meloxicam	- None
**173**	- Flunixine meglumine	- None
**174**	- None	- None
**175**	- Meloxicam	- None
**176**	- Flunixine meglumine	- None
**177**	- Flunixine meglumine	- None
**178**	- None	- Propylene glycol<br>- Vitamin E/Selenium<br>- KCl<br>- Ca
**179**	- None	- None
**180**	- Flunixine meglumine	- None
**181**	- None	- None
**182**	- Ketoprofen	- None
**183**	- None	- None
**184**	- Flunixine meglumine	- Sodium sulfate<br>- Vitamin E/Selenium<br>- KCl
**185**	- None	- None

**186**	- Ketoprofen	- Propylene glycol<br>- Sodium Sulfate<br>- Vitamin E/Selenium<br>- KCl<br>- Ca
**187**	- Flunixine meglumine	- None
**188**	- Ketprofen	- None
**189**	- None	- Sodium sulfate<br>- Vitamin E/Selenium<br>- KCl
**190**	- Flunixine meglumine	- Propylene glycol<br>- Sodium Sulfate<br>- Vitamin E/Selenium<br>- KCl
**191**	- Ketoprofen	- None
**192**	- None	- None
**193**	- Flunixine meglumine	- None
**194**	- None	- None
**195**	- Flunixine meglumine	- None
**196**	- None	- None
**197**	- Ketoprofen	- None
**198**	- Ketoprofen	- None
**199**	- Flunixine meglumine	- None
**200**	- Flunixine meglumine	- None
**201**	- Flunixine meglumine	- None
**202**	- Flunixine meglumine	- None
**203**	- Ketoprofen	- None
**204**	- Flunixine meglumine	- None
**205**	- None	- None
**206**	- None	- None
**208**	- Flunixine meglumine	- None
**209**	- None	- None
